# Anomalous Left Coronary Artery from the Pulmonary Artery Presenting with Atypical Chest Pain in an Adult: A Case Report

**Published:** 2017-07

**Authors:** Reza Jafarzadeh Esfehani, Sara Hosseini, Mahmood Ebrahimi, Majid Jalalyazdi, Azadeh Mahmoudi Gharaee

**Affiliations:** 1 *Department of Medical Genetics, Mashhad University of Medical Sciences, Mashhad, Iran.*; 2 *Medical Genetics Research Center, Mashhad University of Medical Sciences, Mashhad, Iran.*; 3 *Imam Reza Hospital, Mashhad University of Medical Sciences, Mashhad, Iran.*

**Keywords:** *Coronary vessels*, *Coronary angiography*, *Heart defects, congenital*

## Abstract

The anomalous origin of the left coronary artery from the pulmonary artery (ALCAPA) is a rare congenital anomaly. The usual clinical course is severe left-sided heart failure and mitral valve insufficiency presenting during the first months of life. However, in some cases, the collateral blood supply from the right coronary artery is sufficient and symptoms may be subtle or even absent. We describe a 49-year-old woman presenting with atypical chest pain during physical exertion. The exercise tolerance test and then coronary angiography by indication revealed an anomalous origin of the left coronary artery. The patient underwent surgical treatment, whereby a pulmonary artery tube graft from the aorta to the left coronary artery was created and the main pulmonary artery was reconstructed with a bovine pericardial patch. The patient was discharged from the hospital without any chest pain and dyspnea and was symptom free during a follow-up period of 18 months. Clinicians should consider ALCAPA as a differential diagnosis in adults with presentations similar to exercise-related asthma.

## Introduction

The anomalous origin of the left coronary artery from the pulmonary artery (ALCAPA) occurs once per 300000 live births.^1^ Most ALCAPA cases die within the first year of life if left untreated.^2^ Various clinical symptoms may occur in adults with untreated disease. Sudden death is the most common consequence of untreated ALCAPA during the third decade of life.^3^^, ^^4^ Echocardiography might be the first diagnostic tool in determining the origin of the left coronary artery (LCA).^5^ Computed tomography (CT) and magnetic resonance imaging (MRI) are two other diagnostic tools.^6^ Regardless of age or symptoms, early surgical intervention is recommended for all ALCAPA patients.^2^^, ^^3^ In this report, we discuss the presentation, diagnosis, and surgical treatment of ALCAPA in an adult patient presenting with dyspnea and exercise intolerance, having been managed as exercise-induced asthma since childhood. 

## Case Report

**Figure 1:**
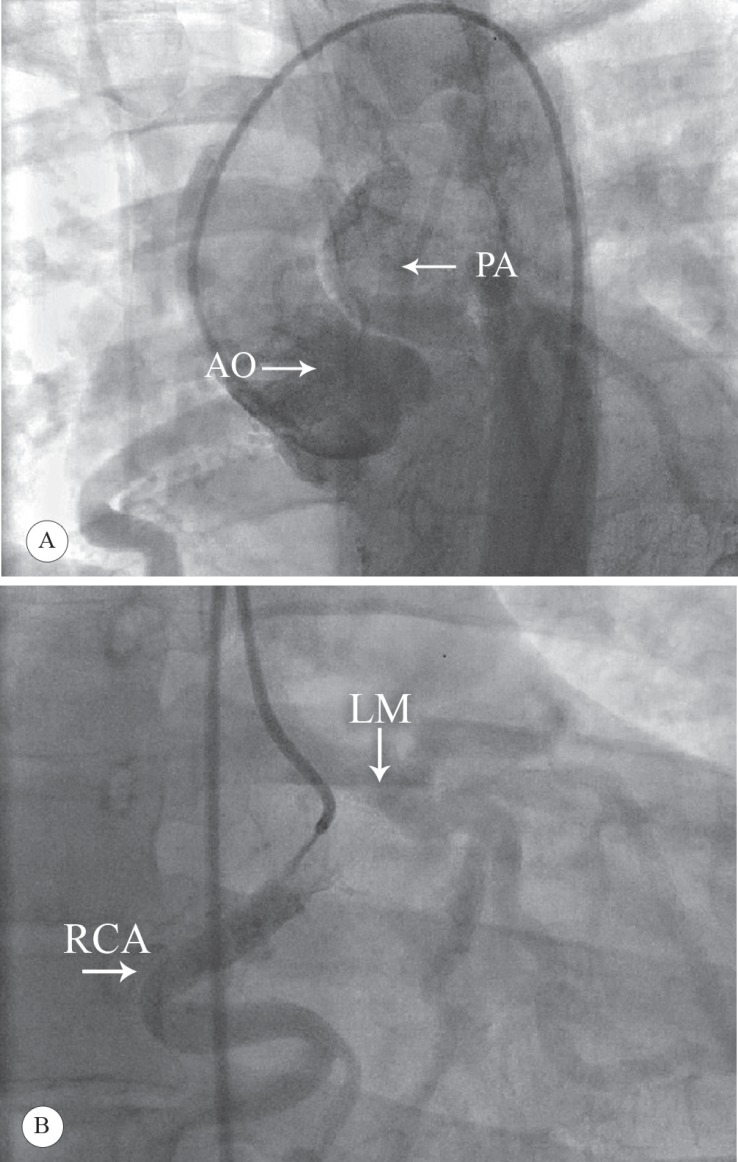
Left anterior oblique angiographic view, illustrates that the pulmonary artery is filled after the aortic root injection from the left coronary system (A). Right anterior oblique angiographic view, shows that the left coronary artery is filled with selective right coronary artery dye injection (B).

RCA, Right coronary artery; LM, Left main; Ao, Aorta; PA, Pulmonary artery

A 49-year-old woman presented with chronic dyspnea on exertion and exercise intolerance. She had been receiving treatment since childhood for presumed exercise-induced asthma. The patient referred to our hospital after experiencing exacerbated left-sided chest heaviness. She had no coronary risk factors and family history of premature coronary artery disease or congenital heart disease. Physical examination was normal except for a subtle systolic murmur best audible at the apex and the lower right sternal border. Electrocardiography (ECG) showed sinus rhythm without ST or T wave changes. Chest X-ray demonstrated no cardiomegaly. Serial cardiac enzymes were negative. In light of the cardiac murmur, an echocardiogram was obtained and revealed mild left ventricular (LV) enlargement with preserved LV contractile function and an ejection fraction of 65%. There were no structural abnormalities of the aortic, mitral, tricuspid, or pulmonic valves. Doppler ultrasound scan revealed mild mitral regurgitation. In the exercise tolerance test, ischemic changes were observed in early stages. A subsequent cardiac catheterization procedure revealed the diagnosis of ALCAPA with a retrograde filling through the collaterals arising from an enlarged right coronary artery (RCA) (12 mm) (Figure 1), pronounced left-to-right shunting from the LCA into the left main pulmonary artery trunk, and the RCA giving rise to collaterals to a large left anterior descending artery, which was ectatic in its proximal segment, into a smaller circumflex artery. The patient underwent surgical treatment, whereby a pulmonary artery tube graft was inserted from the aorta into the LCA and the main pulmonary artery was reconstructed with a bovine pericardial patch. The patient had no major complications or ischemic symptoms 18 months after the operation. A follow-up echocardiogram showed normalized stroke volume and left atrial and ventricular size secondary to reversed left-to-right shunting, preserved LV ejection fraction at 55%, and absence of valvular abnormalities.

## Discussion

ALCAPA accounts for approximately 0.25-0.5% of congenital heart diseases.^7^ The diagnosis of ALCAPA in adults is growing due to the recent developments in diagnostic tools such as cardiac MRI and ECG-gated multidetector CT angiography. The most common findings of the adult cases of ALCAPA are the irreversible impairment of the cardiac function, sudden cardiac death, acute myocardial infarction, malignant arrhythmias secondary to myocardial scar tissue, impaired LV contractile function, and development of significant mitral regurgitation.^3^ In our patient, regardless of the late presentation, a preserved LV contractile function without apparent structural or functional cardiac impairment may have protected her against a life-threatening event later in life. It is known that a sufficient collateral blood supply from the RCA could help some patients with ALCAPA pass through childhood with relatively minor symptoms, including dyspnea, chest pain, and exercise intolerance. These symptoms are often misinterpreted, as was the case in our patient, as exercise-induced asthma. Adult cases mostly present with the symptoms of angina, palpitation, or fatigue.^3^ The key to the early diagnosis of ALCAPA and preventing permanent cardiac damage is a careful physical and cardiac examination (heart murmur) and clinical suspicion in children with exercise intolerance.^3^

The early employment of the first-line diagnostic modality, the exercise tolerance test, is essential bearing in mind that ECG is usually unrevealing. The diagnostic hallmark of ALCAPA is visualizing the LCA artery arising from the main pulmonary artery.^6^ The prominent dilatation of the RCA and LCA may develop over time because of the shunted blood flow from the RCA to the LCA and to the main pulmonary artery.^6^ There is also a retrograde flow from the LCA to the main pulmonary artery.^6^ Another finding is myocardial ischemia, which leads to the dilatation and hypertrophy of the LV.^6^ These findings can be visualized by echocardiography, CT, or MRI. Before the development of CT and MRI, an invasive technique such as angiography was used to diagnose ALCAPA.^3^ The presence of an increased systolic coronary flow in transthoracic pulse wave Doppler is a unique diagnostic parameter in ALCAPA cases with a collateral circulation and a coronary left-to-right shunt.^8^ The parasternal pulmonary artery short-axis view in echocardiography can visualize the anomalous origin of the LCA.^5^ Although CT angiography offers better spatial resolution than MR imaging, it cannot asses the blood flow and has a high radiation dose.^6^ ECG-gated multidetector CT angiography is a valuable first-line modality for assessing ALCAPA and a good follow-up tool in adults.^6^

Several surgical modalities have been proposed for the treatment of ALCAPA. A surgical option is the ligation of the anomalous artery at its origin in order to prevent the coronary steal phenomena, but this method depends on an extensive collateral supply from the RCA. Today, surgical procedures are aimed at creating a two-coronary system either via a bypass graft in combination with the ligation of the anomalous artery or via the Takeuchi procedure, whereby an intrapulmonary tunnel from the aortopulmonary window to the coronary artery is created, or via the translocation of the LCA from the pulmonary trunk to the aortic sinus.^9^^-^^11^ The latter depends on the distance between the origin of the anomalous artery and the aorta but is possible in the majority of the cases. However, ALCAPA can be managed without surgery in cases where surgery is not applicable. Tian et al.^12^ reported ALCAPA in an adult patient, who refused to undergo surgery and was, subsequently, treated with metoprolol (50 mg per day). Their case presented with a continuous murmur (grade 3/6) along the left sternal border, accompanied by a dilated and tortuous RCA arising from the sinus of the aorta and the anomalous origin of the LCA from the pulmonary artery with the presence of multiple collateral vessels between the anterior descending artery and the RCA. The authors reported no record of myocardial ischemia within 3 years of follow-up. 

We performed surgical correction via the Takeuchi procedure on our adult patient with ALCAPA because of the ischemic changes observed in the early stages of the exercise tolerance test and the risk of ischemic cardiomyopathy. The early and late results at 18 month’s follow-up were good. 

## Conclusion

Adulthood ALCAPA should be considered a differential diagnosis in adult patients with minor symptoms of exercise intolerance or dyspnea. Therefore, a high clinical suspicion and detailed physical and diagnostic evaluations are required in such cases since timely surgical treatment usually confers an excellent prognosis. 

## Notes:

***This paper should be cited as: ***Jafarzadeh Esfehani R, Hosseini S, Ebrahimi M, Jalalyazdi M, Mahmoudi Gharaee A. Anomalous Left Coronary Artery from the Pulmonary Artery Presenting with Atypical Chest Pain in an Adult: A Case Report. J Teh Univ Heart Ctr 2017;12(3):128-130.
